# Supplementary material for: Complex chromosomal neighborhood effects determine the adaptive potential of a gene under selection
Source: eLife. 2017 Jul 25;6:e25100. doi: 10.7554/eLife.25100 (PMC5526668; doi:10.7554/eLife.25100)

Population trajectories – Legend

Every page shows experimental data from 95 replicate populations (wells A02-H12) and a medium-only control well (A01) of one evolution experiment performed in 96-well plates. Thick black lines = OD_{600} across 10 days plotted on the left y-axis. Dark yellow and blue thick lines = OD_{600}-normalized YFP and CFP fluorescence plotted on the right y-axis. The right y-axis is scaled differently on every page to accommodate maximum values. Ranges of y-axes are shown at the top right corner of every page. The range of x-axes is 1 to 10 days. Thin solid horizontal lines = OD_t, the threshold OD_{600} used to classify populations as rescued. Thin dashed black horizontal lines = threshold fluorescence used to classify populations as high in YFP or CFP fluorescence is shown as a thin dashed black line.

Strain A (IS-wt, Replicate Set 1)

left y-axis [0 1], right y-axis [0 14]

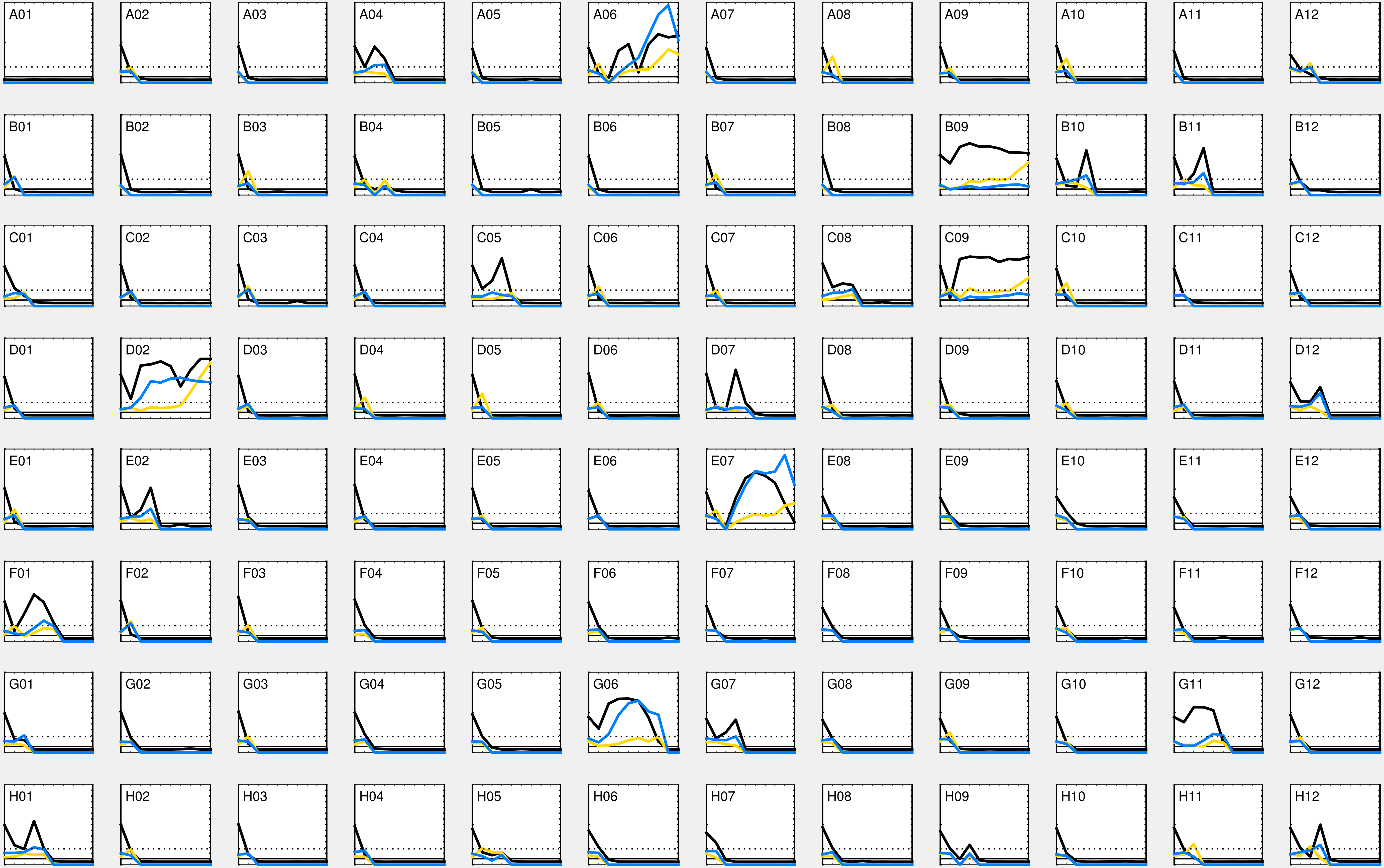

Strain A (IS-wt, Replicate Set 2)

left y-axis [0 1], right y-axis [0 30]

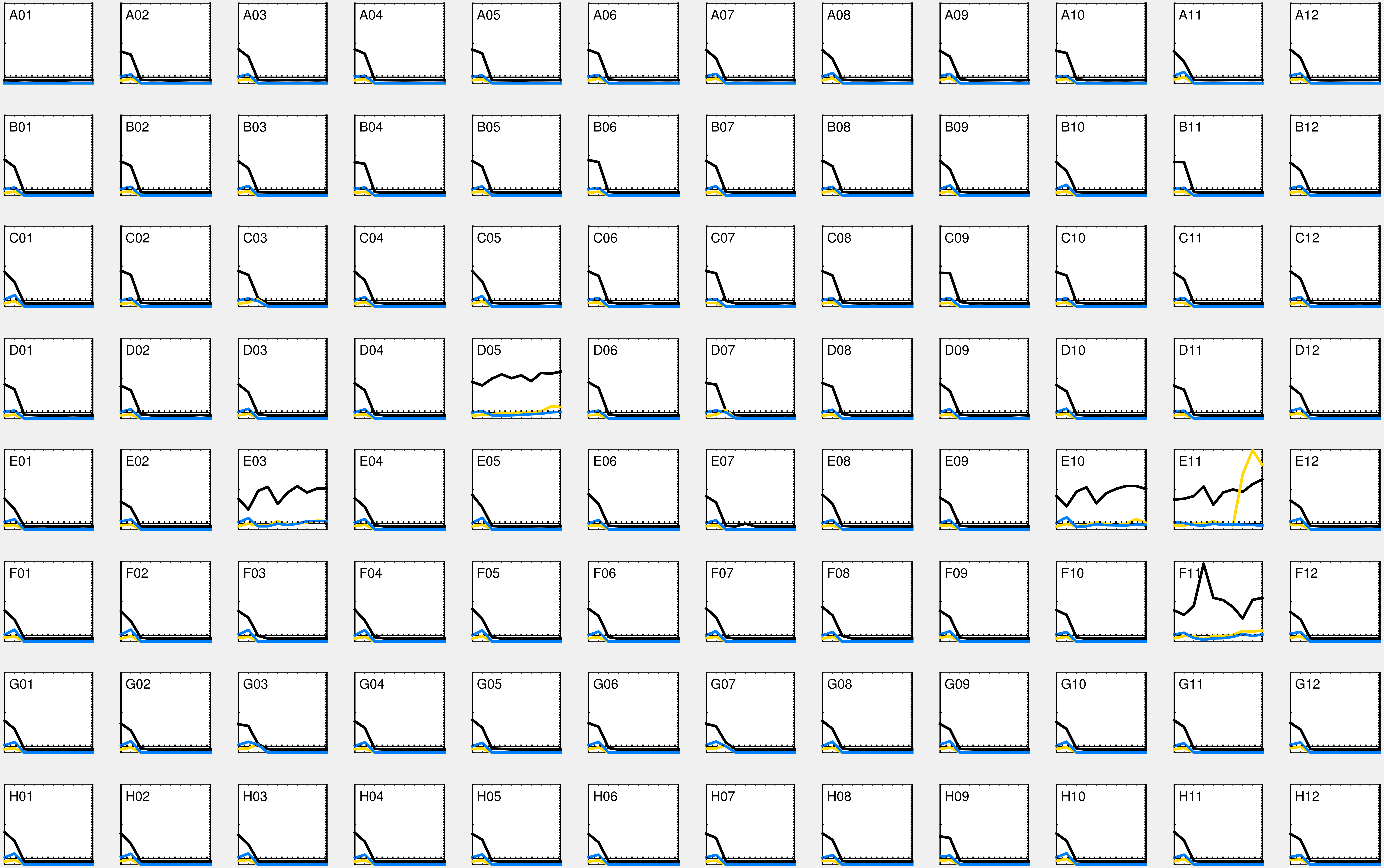

Strain A (IS-wt, Replicate Set 3)

left y-axis [0 1], right y-axis [0 7]

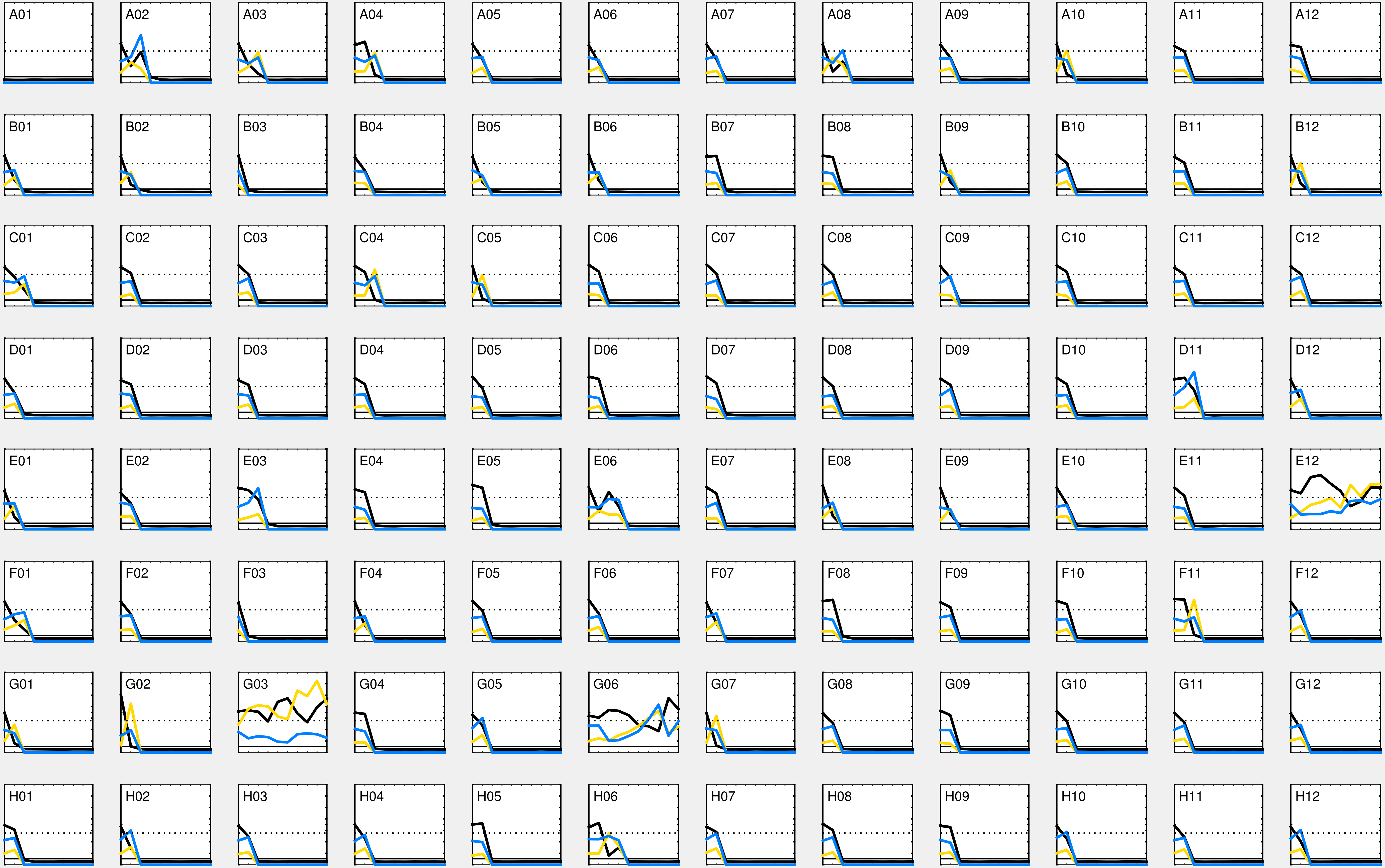

Strain B (IS-wt, Replicate Set 1)

left y-axis [0 1], right y-axis [0 17]

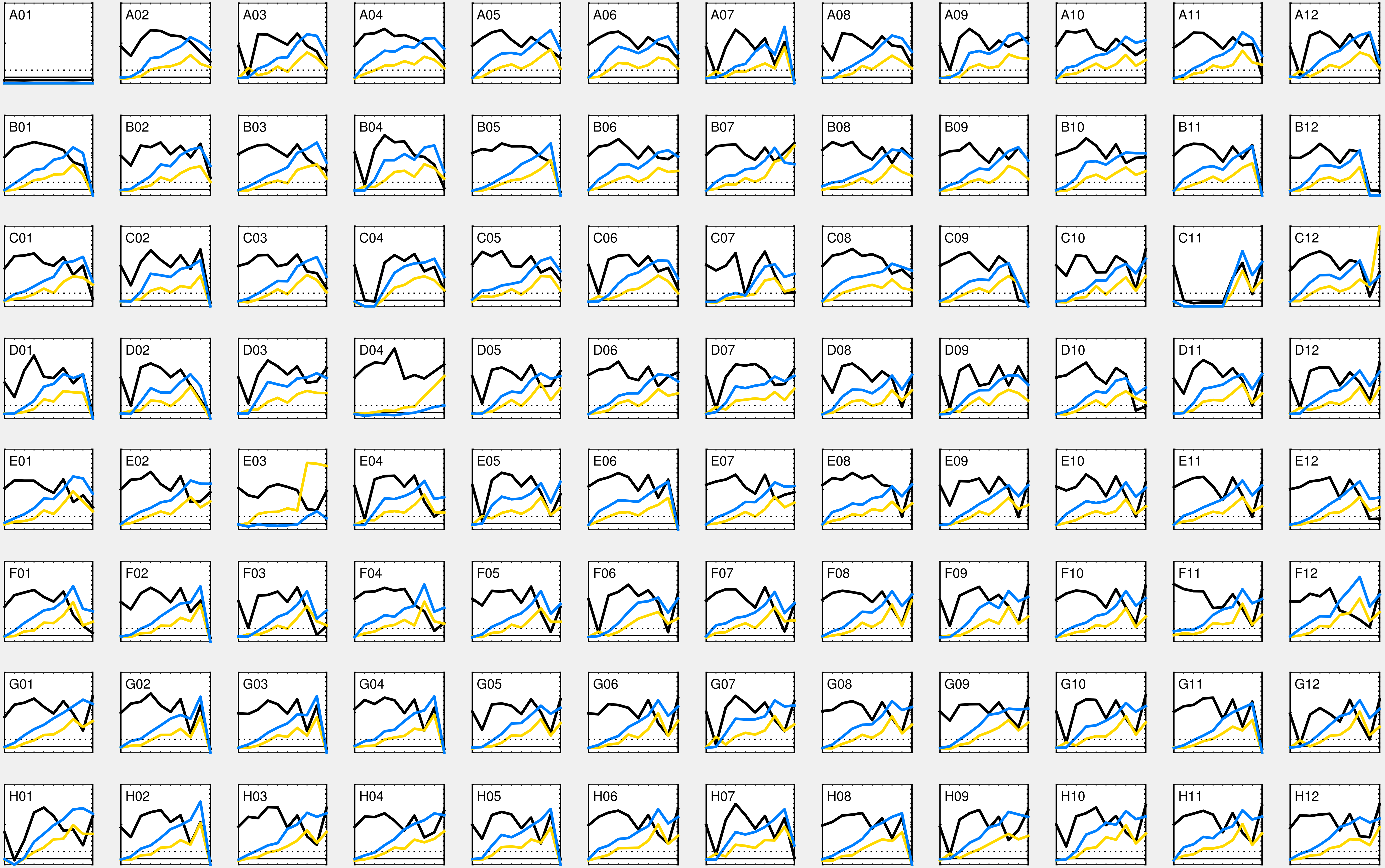

Strain B (IS-wt, Replicate Set 2)

left y-axis [0 1], right y-axis [0 30]

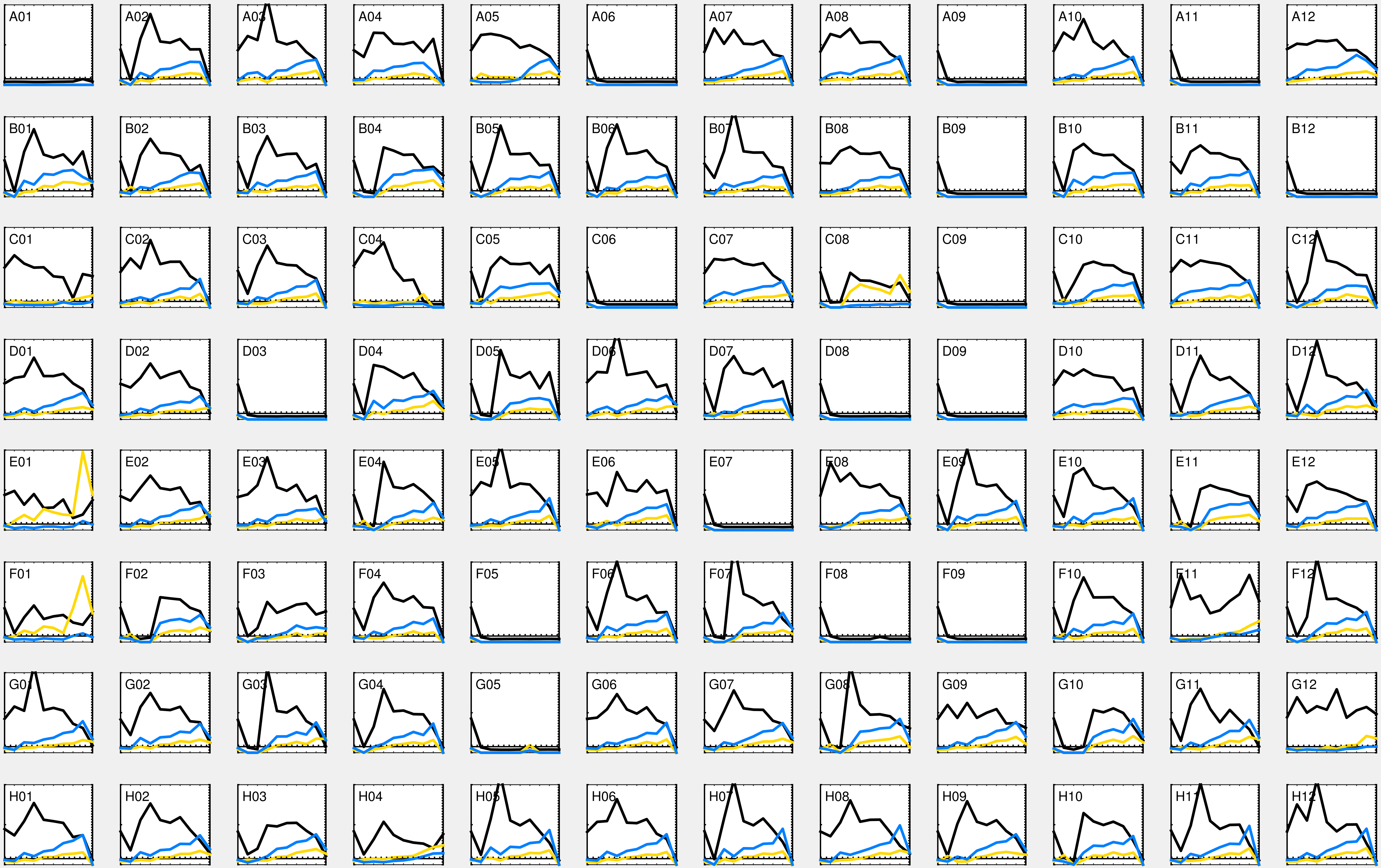

Strain B (IS-wt, Replicate Set 3)

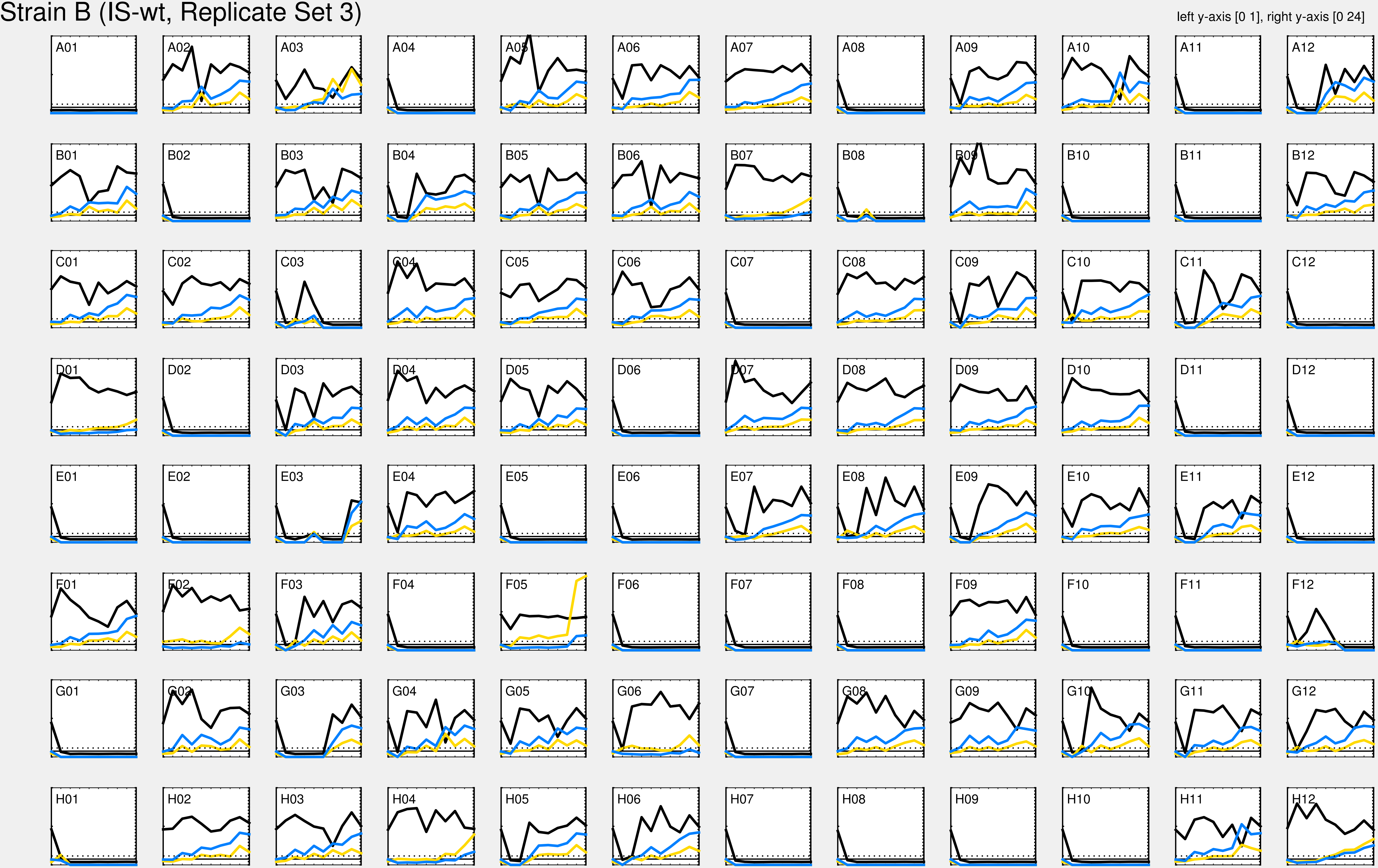

Strain C (IS-wt, Replicate Set 1)

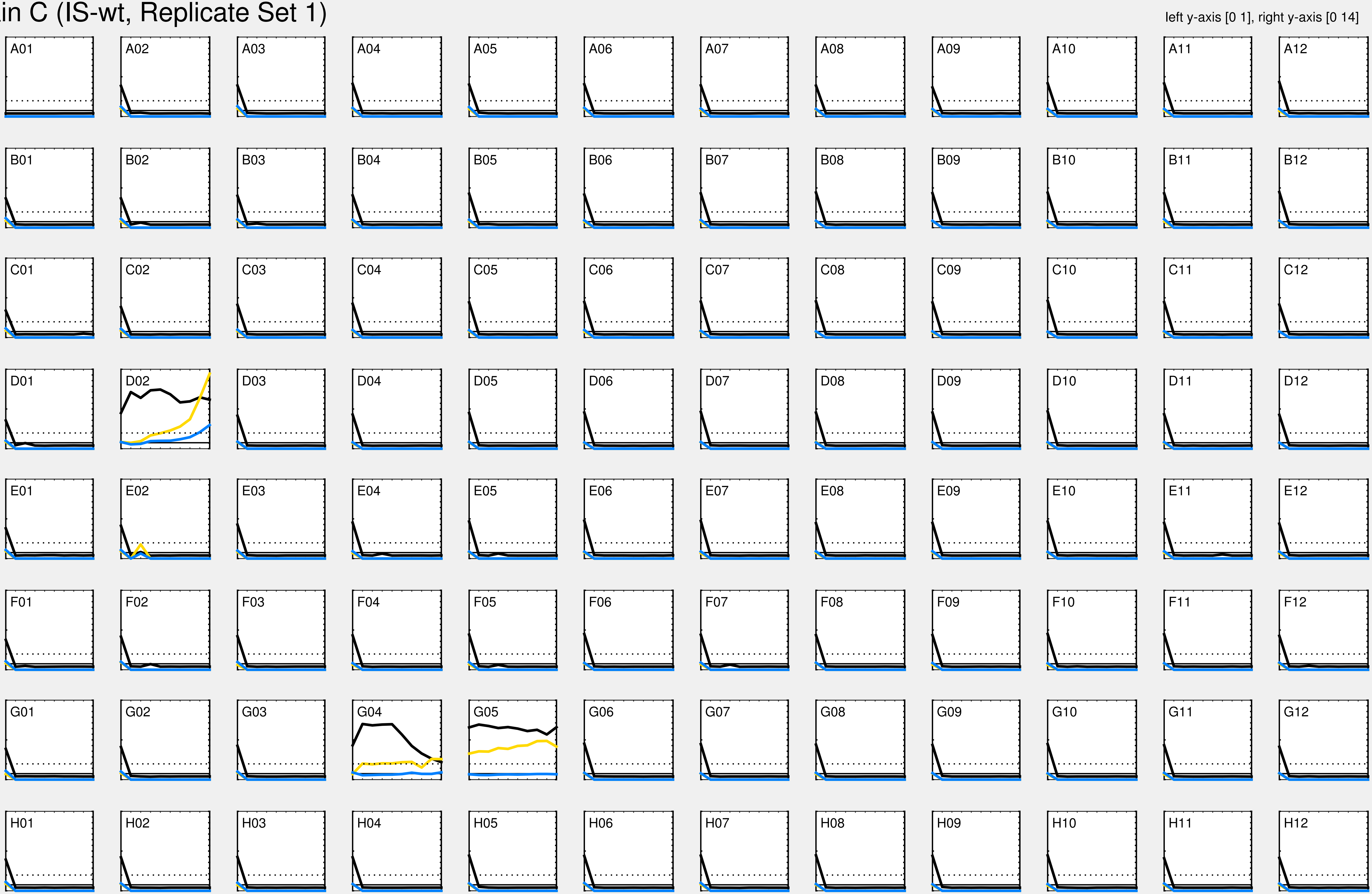

Strain C (IS-wt, Replicate Set 2)

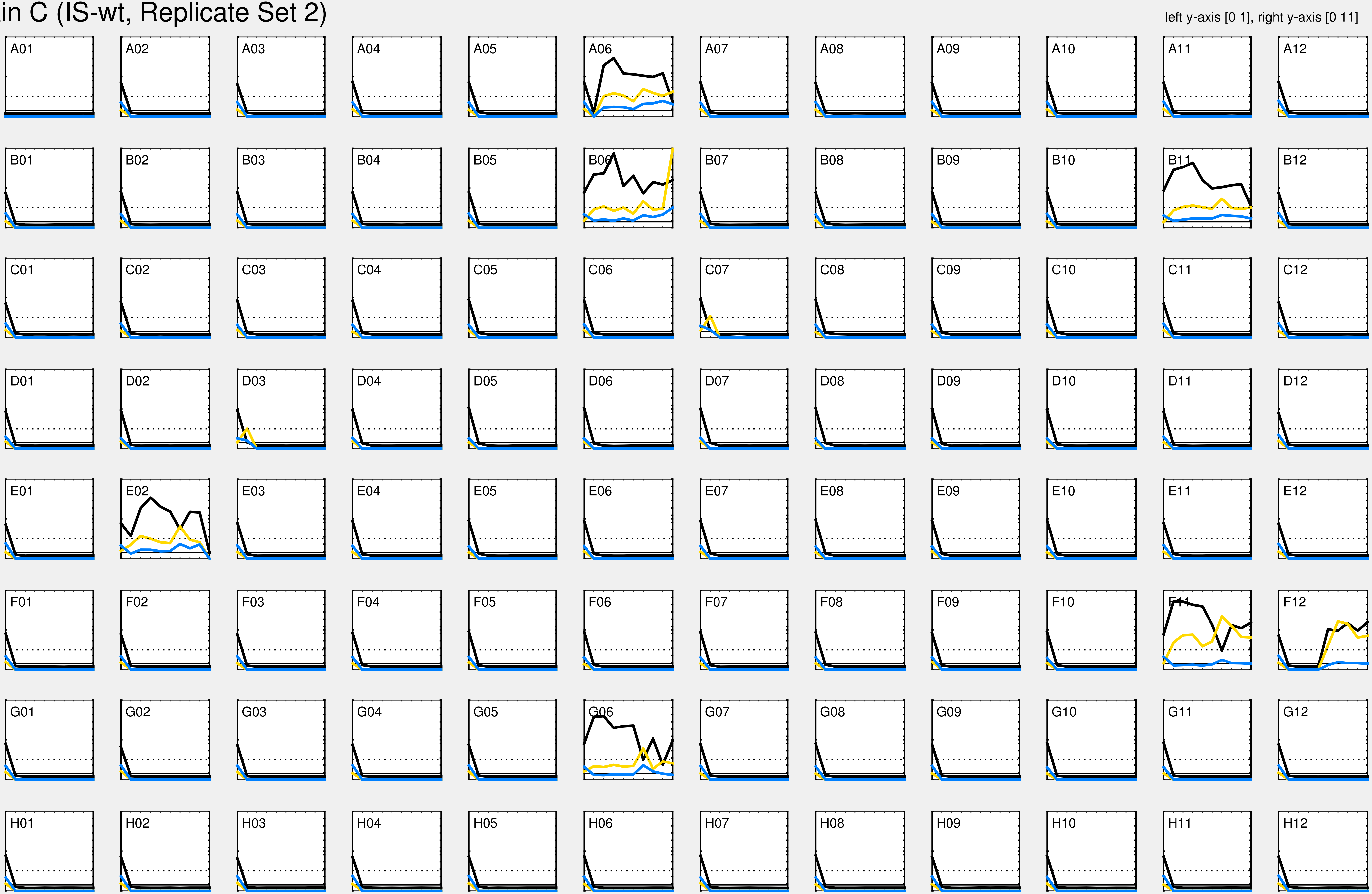

Strain C (IS-wt, Replicate Set 3)

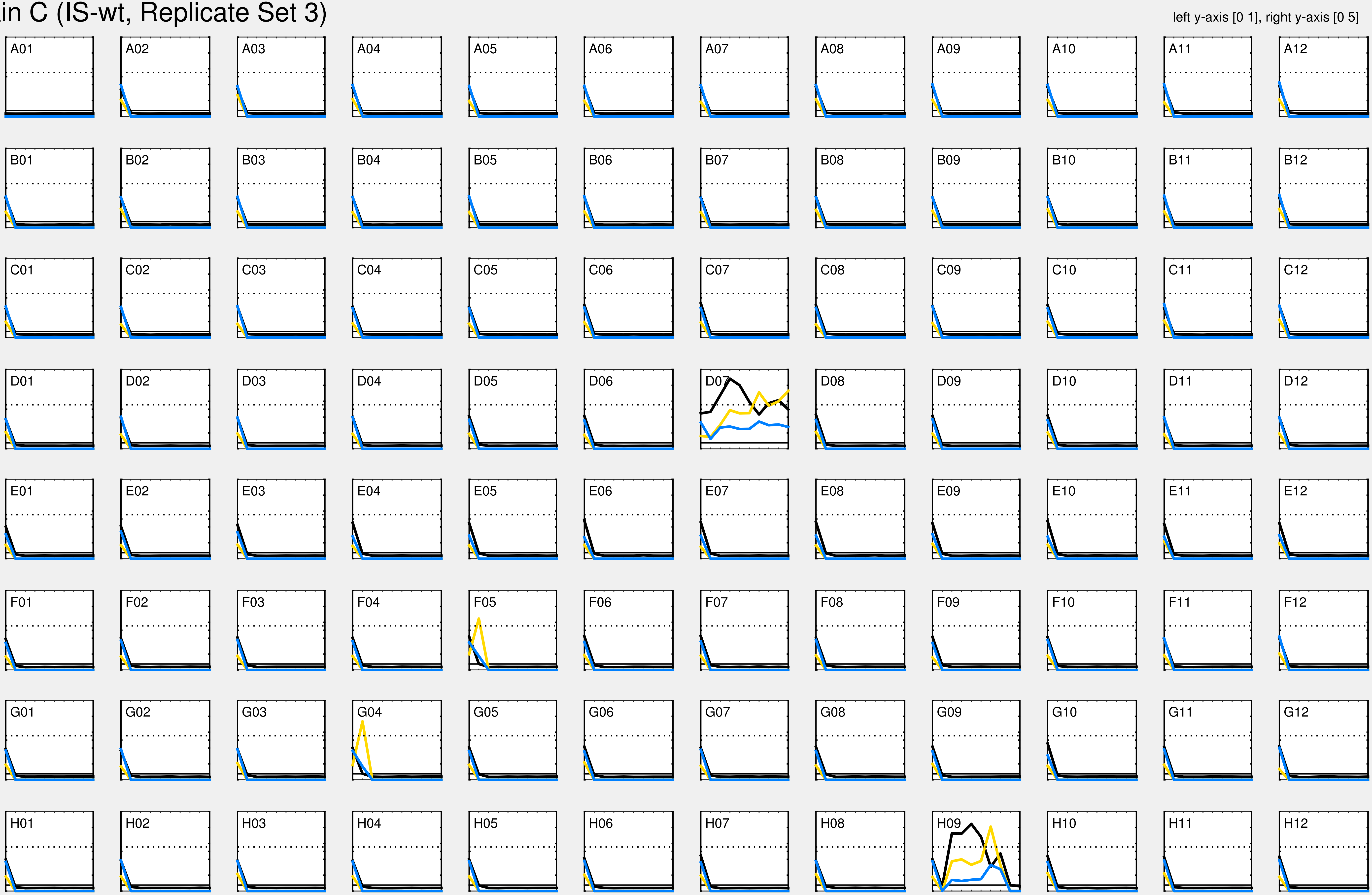

Strain D (IS-wt, Replicate Set 1)

left y-axis [0 1], right y-axis [0 17]

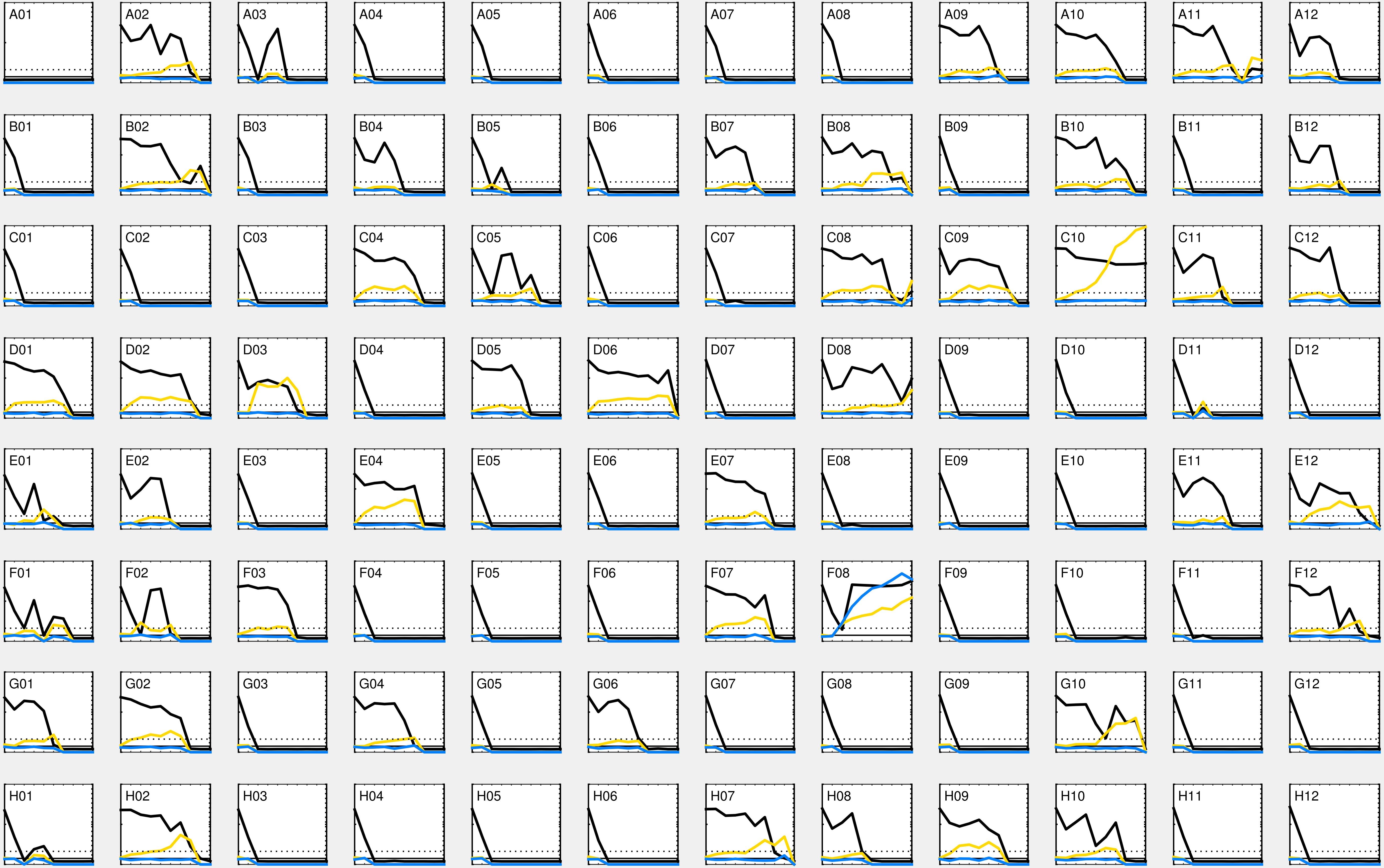

Strain D (IS-wt, Replicate Set 2)

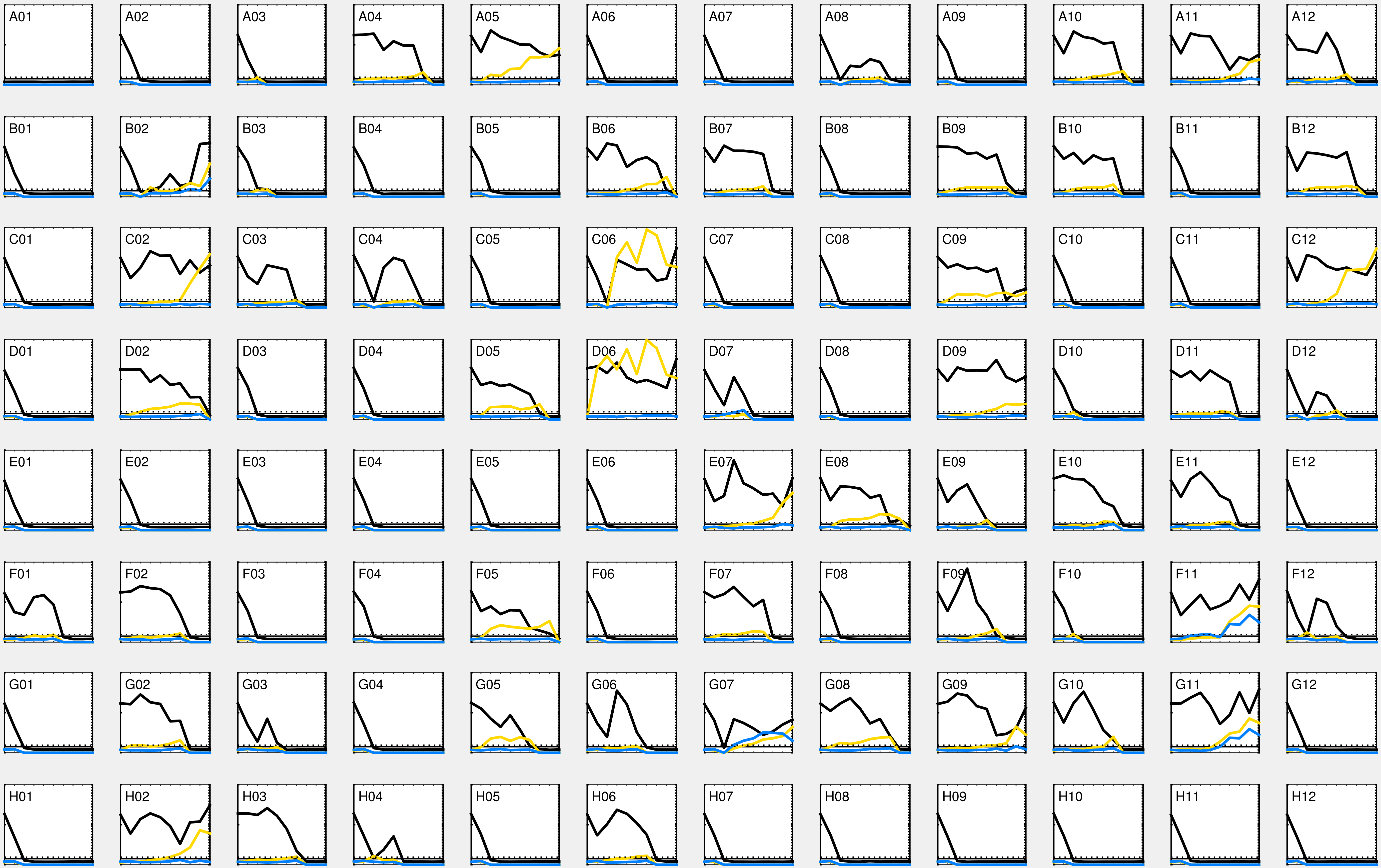

Strain D (IS-wt, Replicate Set 3)

left y-axis [0 1], right y-axis [0 50]

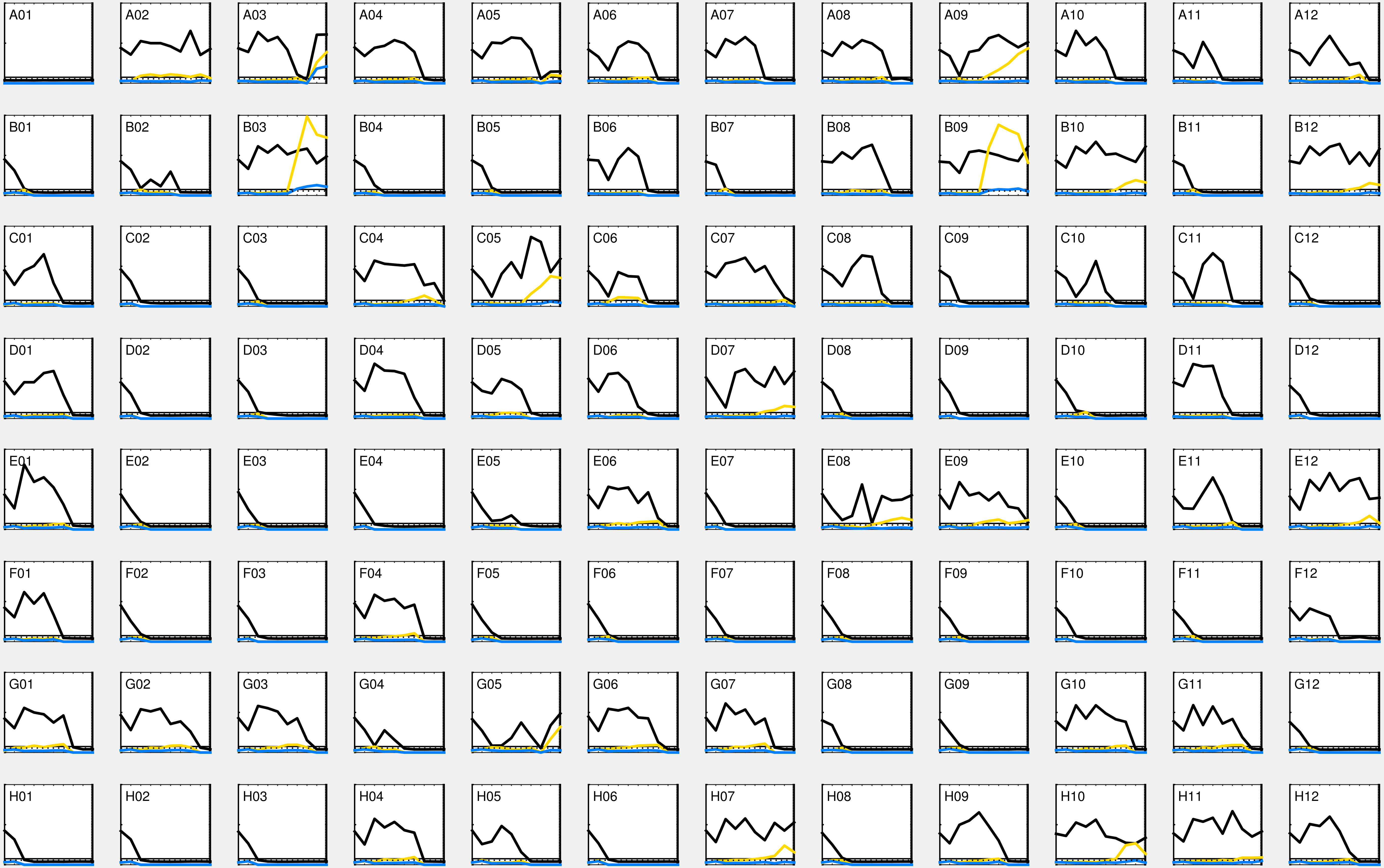

Strain B (IS5I deleted)

left y-axis [0 1], right y-axis [0 34]

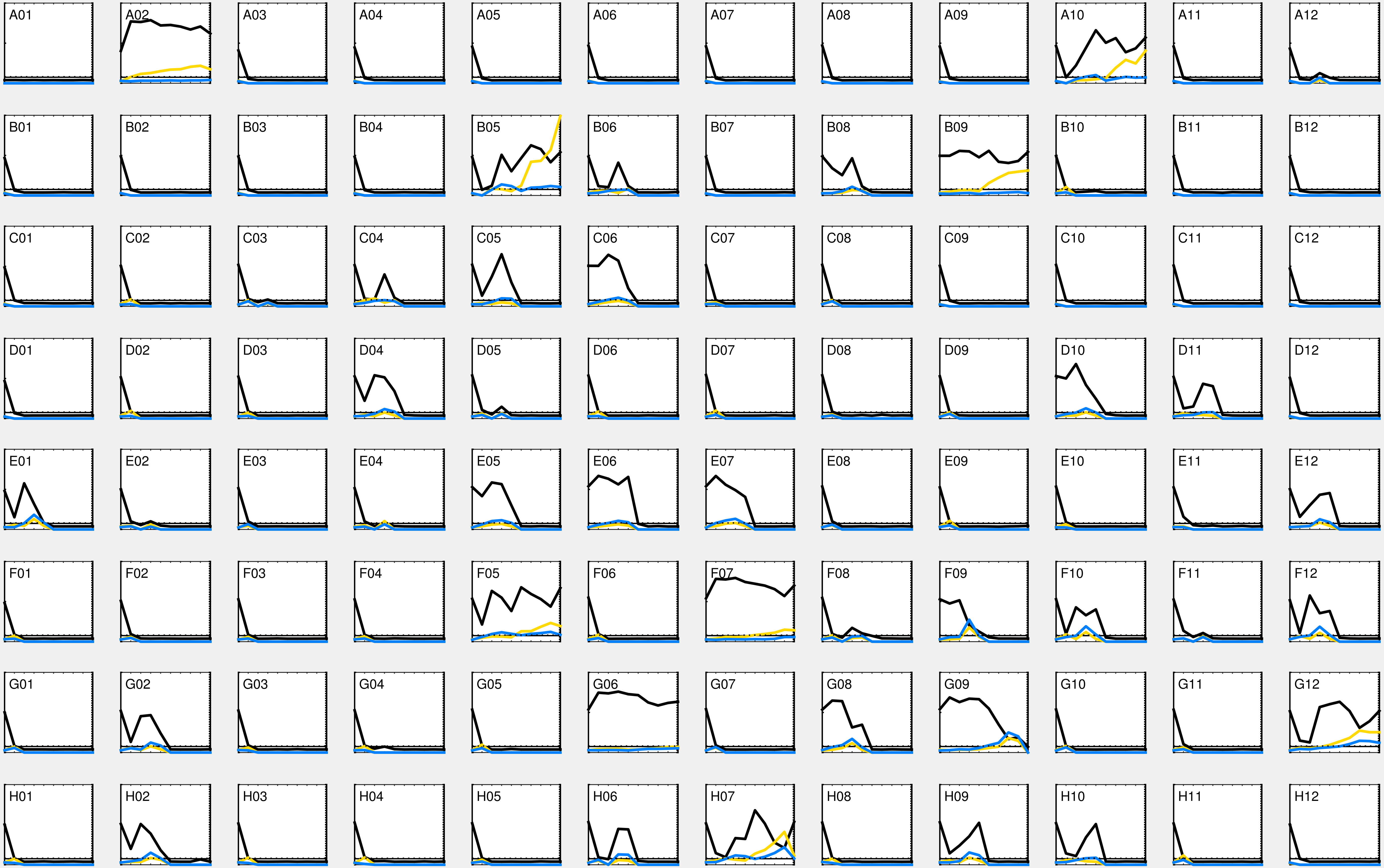

Strain A (IS-free)

left y-axis [0 1], right y-axis [0 3]

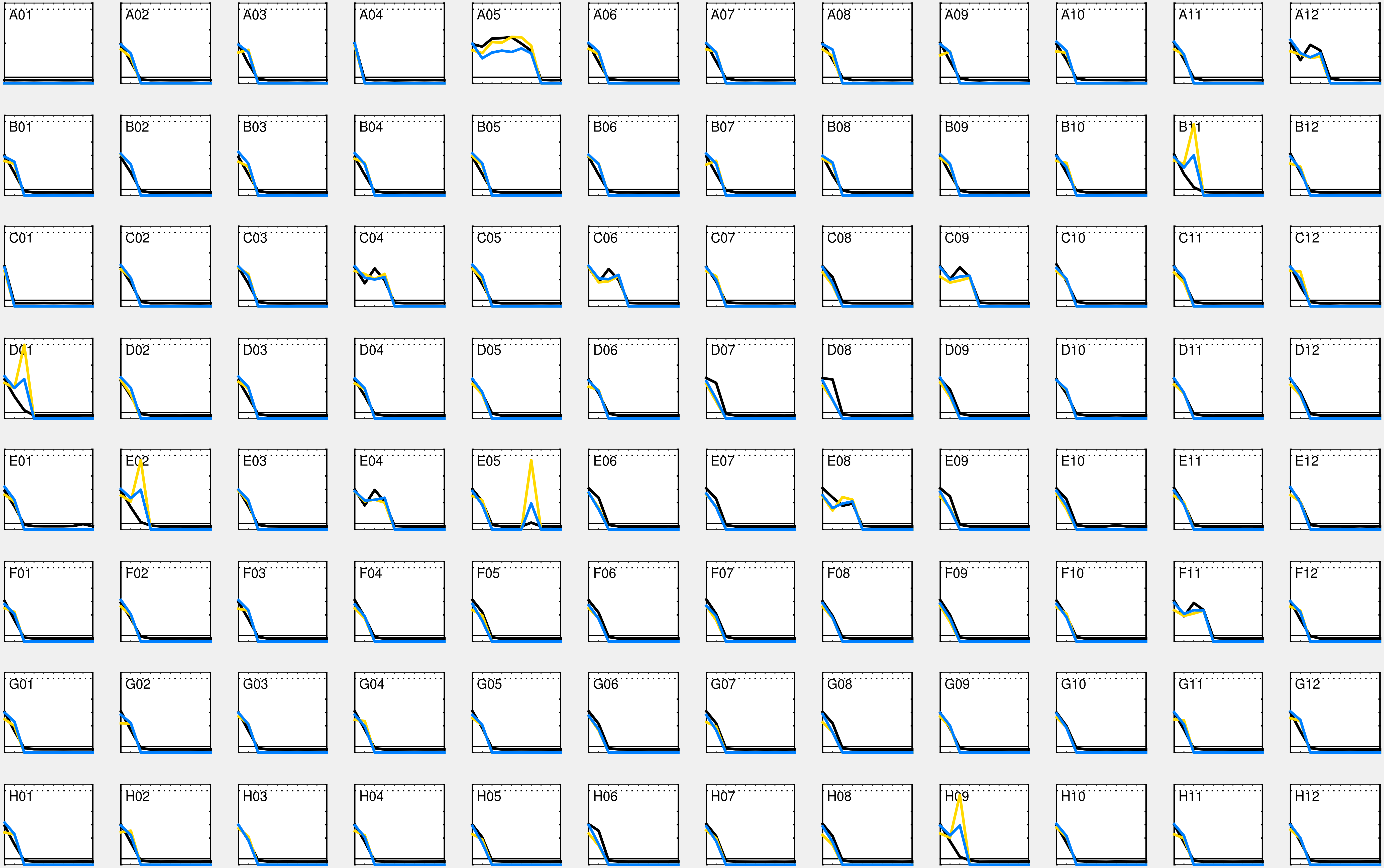

Strain B (IS-free)

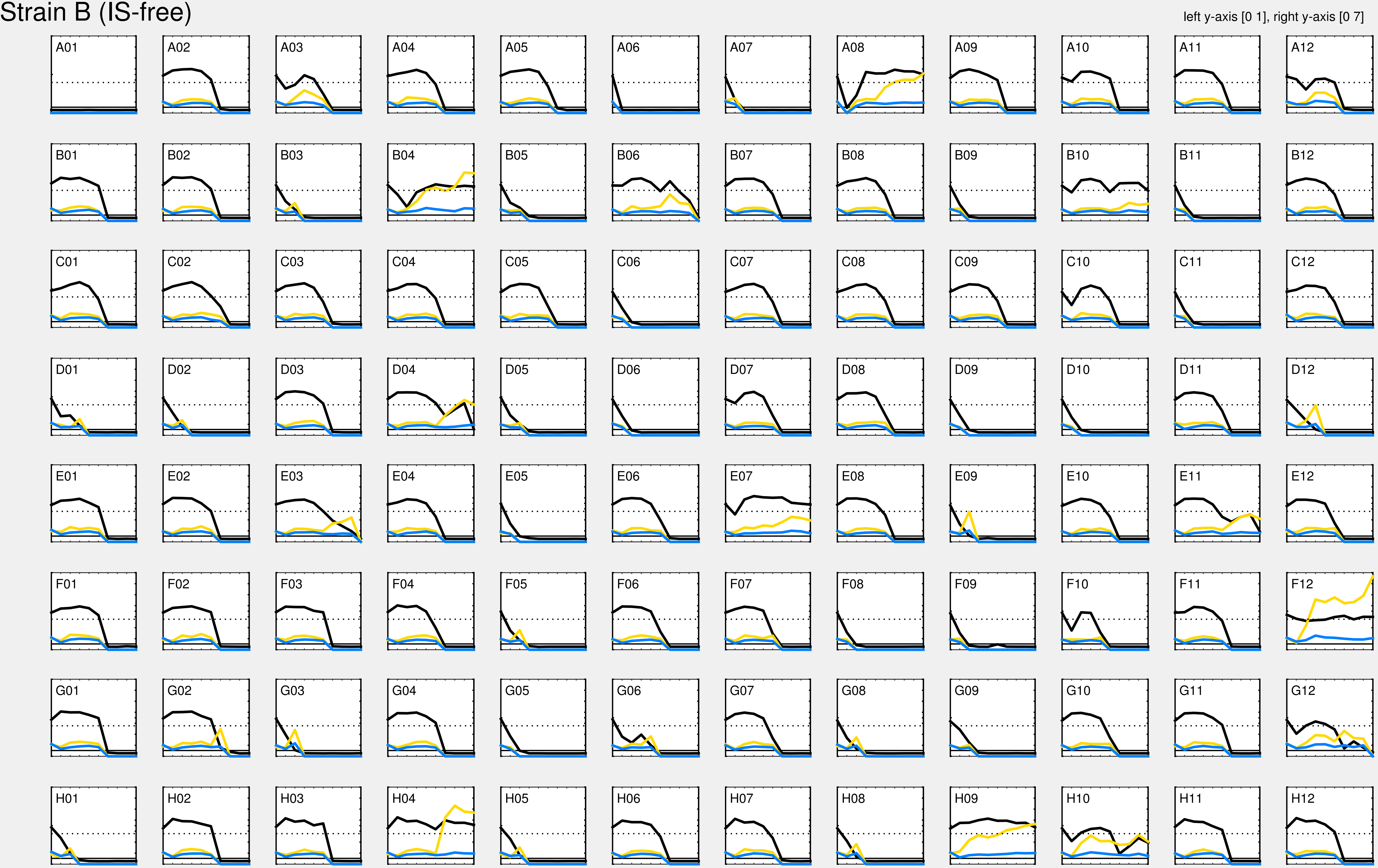

Strain C (IS-free)

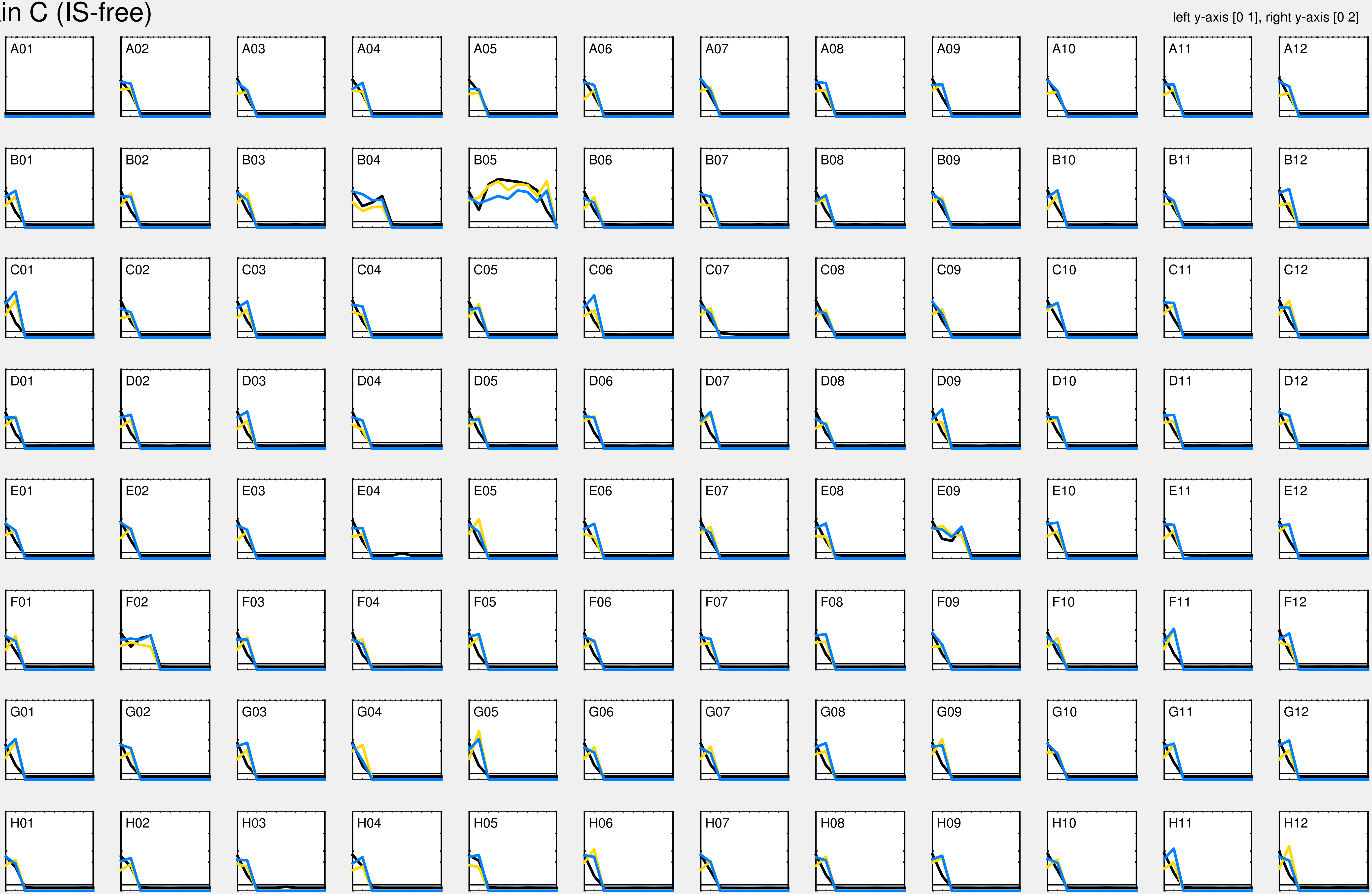

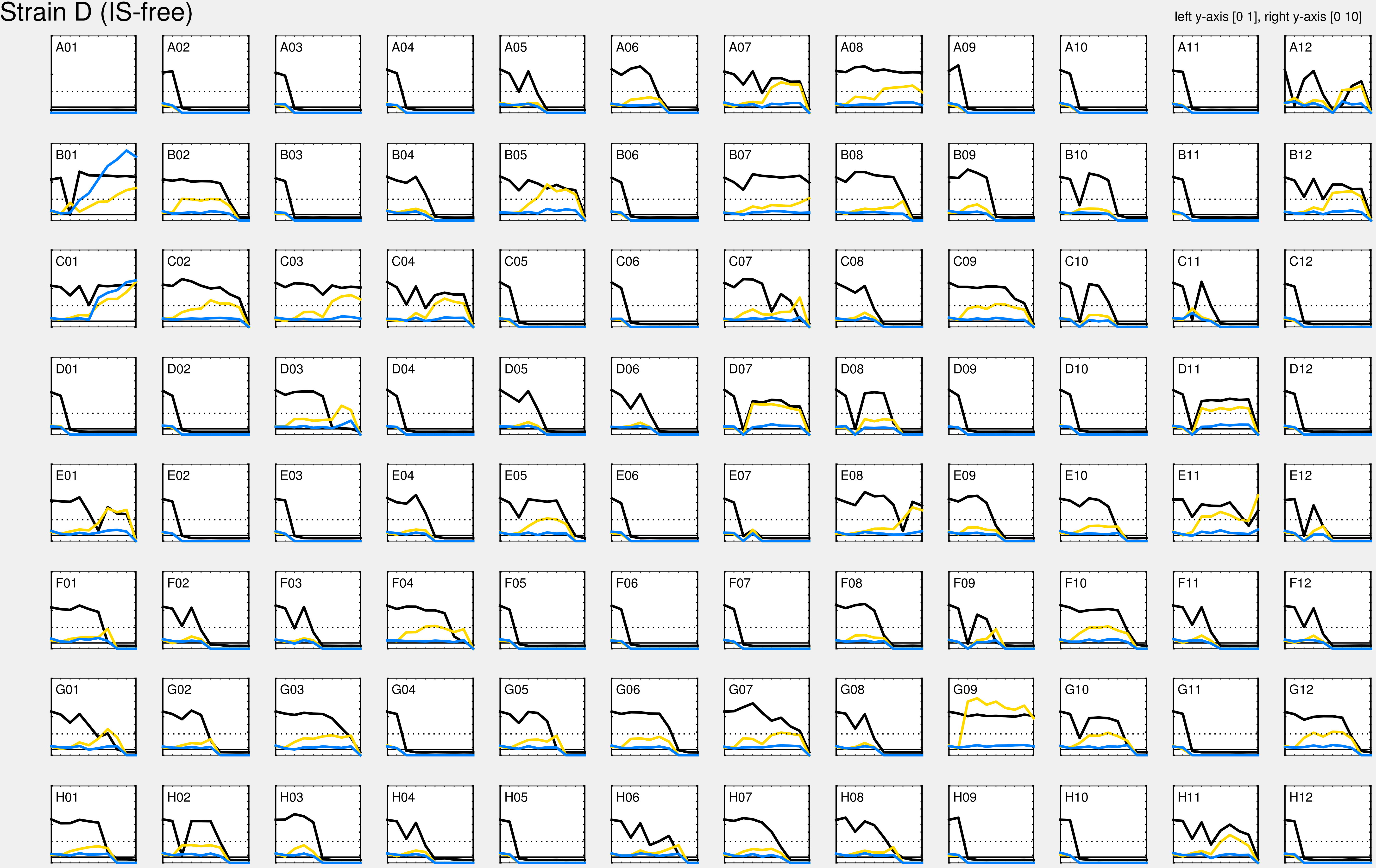
Strain D (IS-free)
left y-axis [0 1], right y-axis [0 10]
A01
A02
A03
A04
A05
A06
A07
A08
A09
A10
A11
A12
B01
B02
B03
B04
B05
B06
B07
B08
B09
B10
B11
B12
C01
C02
C03
C04
C05
C06
C07
C08
C09
C10
C11
C12
D01
D02
D03
D04
D05
D06
D07
D08
D09
D10
D11
D12
E01
E02
E03
E04
E05
E06
E07
E08
E09
E10
E11
E12
F01
F02
F03
F04
F05
F06
F07
F08
F09
F10
F11
F12
G01
G02
G03
G04
G05
G06
G07
G08
G09
G10
G11
G12
H01
H02
H03
H04
H05
H06
H07
H08
H09
H10
H11
H12

Strain E

left y-axis [0 1], right y-axis [0 27]

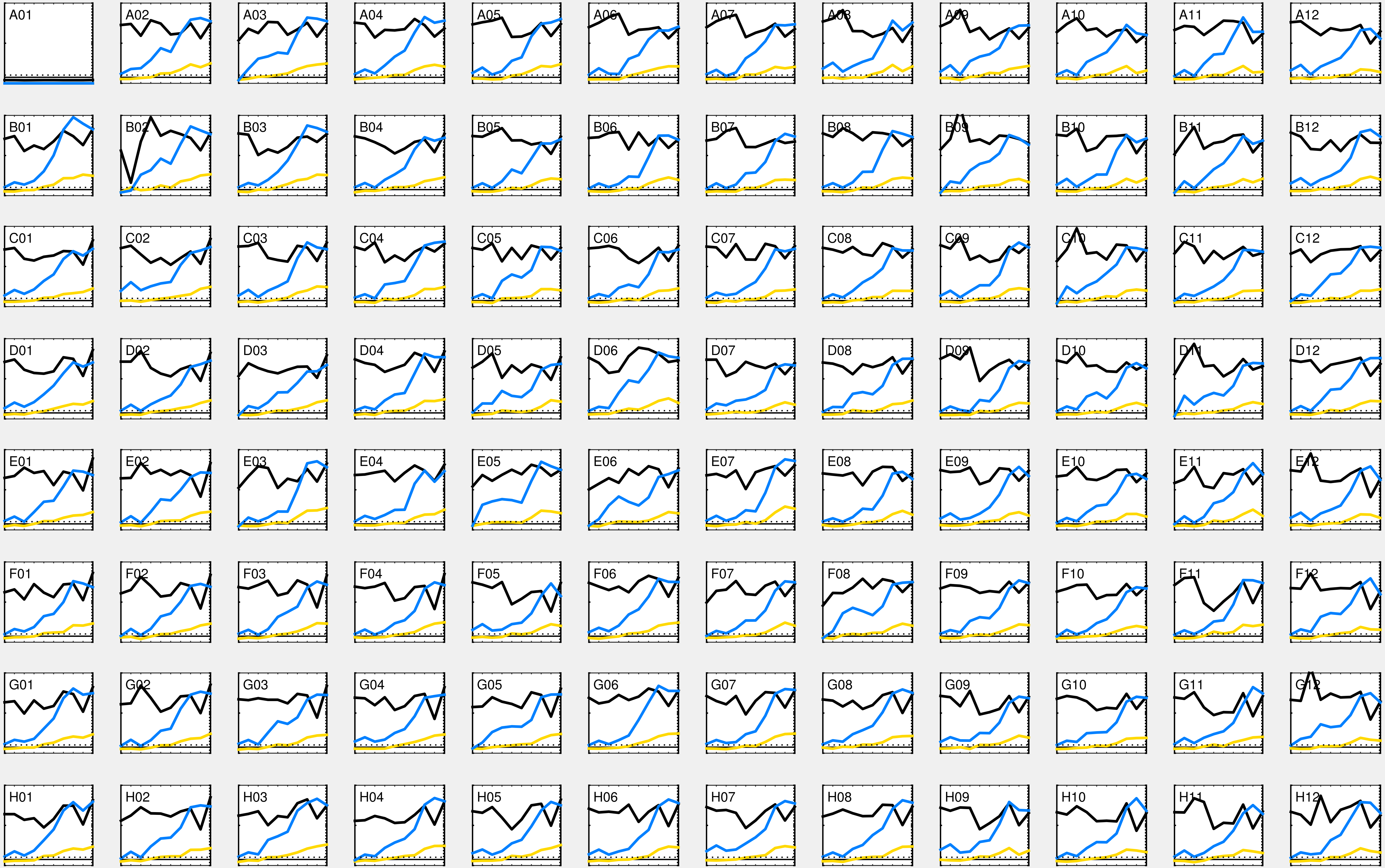

Supplement: Supplementary file 1. — Set of 96-panel figures showing OD and OD-normalized fluorescence values for each population in each of 18 evolution experiments. DOI: http://dx.doi.org/10.7554/eLife.25100.036 [file elife-25100-supp1.pdf]
